# Case report: Echocardiographic diagnosis of double orifice mitral valve in an asymptomatic woman

**DOI:** 10.3389/fcvm.2022.1091201

**Published:** 2023-01-11

**Authors:** Zixian Deng, Xiaoyu Wang, Qiyun Liu, Jianghua Li, Huadong Liu

**Affiliations:** ^1^Department of Cardiology, Shenzhen People's Hospital, Second Clinical Medical College of Jinan University, First Affiliated Hospital of Southern University of Science and Technology, Shenzhen, Guangdong, China; ^2^Department of Cardiology, Shenzhen Cardiovascular Minimally Invasive Medical Engineering Technology Research and Development Center, Shenzhen People's Hospital, The Second Clinical Medical College, The First Affiliated Hospital, Southern University of Science and Technology, Jinan University, Shenzhen, China

**Keywords:** congenital heart disease, case report, double orifice mitral valve, diagnosis, echocardiography

## Abstract

Double orifice mitral valve (DOMV) is a rare congenital anomaly that is often associated with cardiac malformation. Valve dysfunction usually presents in childhood; therefore, most cases are diagnosed with DOMV in childhood. Its prevalence and prognostic relevance in adulthood are unknown. Here, we report a case of a 38-year-old woman who presented to the outpatient clinic with an abnormal electrocardiogram and was found to have an isolated double orifice mitral valve malformation on transthoracic echocardiography. Echocardiography is the diagnostic tool of choice for patients with double orifice mitral valves. We should familiarize ourselves with the echocardiographic features of DOMV in order to improve the detection.

## Introduction

Double orifice mitral valve (DOMV) is an extremely rare congenital anomaly that may be due to inadequate fusion of the endocardial cushions during embryonic life, resulting in two separate orifices of the mitral valve into the left ventricle, and can be classified into 3 types: complete bridging, incomplete bridging, and hole type (1). Other structural cardiac abnormalities are usually associated with them and isolated DOMV is rarely present (2). Here, we report an incomplete bridging type of DOMV in an asymptomatic woman.

## Case presentation

**Figure 1:**
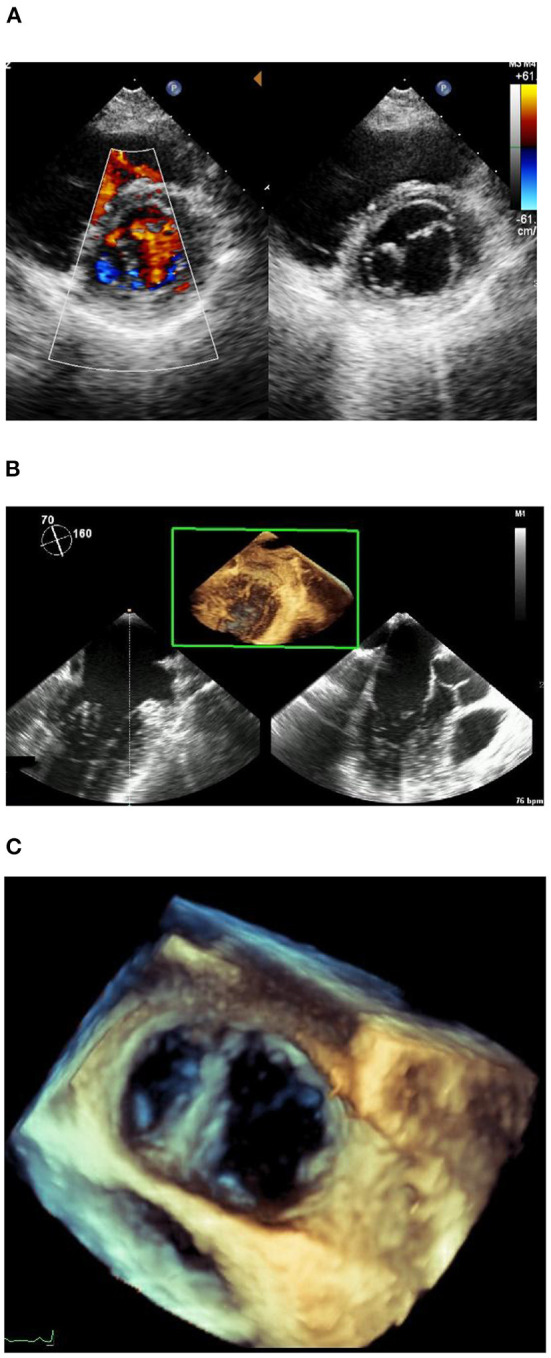
**(A)** Short axis of echocardiogram image. **(B)** Transesophageal echocardiogram. **(C)** Transesophageal echocardiogram.

A 38-year-old asymptomatic woman with a previous history of subclinical hypothyroidism and long-term use of sodium thyroxine presented to the outpatient clinic with “abnormal ECG for 1 year.” No heart murmur was detected. The short axis view showed two oval-shaped orifices, one large and one small, aligned left and right, with the small hole tendon not visible (Figure 1A). Color Doppler examination showed two bundles of blood flow across the orifice. There was no mitral stenosis (MS) and trivial mitral regurgitation (MR) (peak mitral E wave velocity = 97.2 cm/s, peak gradient = 4 mm Hg, peak mitral A wave velocity = 47.4 cm/s, peak gradient = 1 mm Hg). The remaining valvular structures were not significantly abnormal, and the tricuspid valve had mild regurgitation. The left ventricular ejection fraction and diastolic function were normal. In addition, no other cardiac structural abnormalities were detected. The transesophageal echocardiogram (Figures 1B, C) was consistent with the transthoracic echocardiogram, and the diagnosis of congenital heart disease was confirmed as DOMV. Additional movies are available in the Supplementary material.

## Discussion

Double orifice mitral valve is an extremely rare congenital anomaly, first reported by Greenfield (3). The etiology of this disease remains unclear. Some scholars believe that DOMV may result from the inadequate fusion of the endocardial cushions during embryonic development (4). Others suggest that it may be related to interstitial abnormalities and atrioventricular sulcus folding abnormalities during endocardial cushion formation in valve tissue or abnormal development of the ventricular myocardium (5). But a small portion is acquired, and when mitral annular calcifications affect the second scallop of the anterior and the posterior leaflet, the mitral valve orifice may split into two, leading to DOMV (6). In addition, iatrogenic interventions are also potential causes of DOMV. As described by Alfieri, DOMV can occur after “edge-to-edge” mitral valve repair, a technique that specifically refers to the suturing together with the middle scallops of the mitral leaflets, which then form a double-barrel opening (7, 8). Due to its rarity, epidemiological data are lacking. Previous retrospective studies and autopsy reports have found the prevalence of DOMV to be between 0.01 and 0.1% (1, 9, 10).

The anatomic feature of DOMV is an accessory bridge of fibrous tissues extending from the center of the posterior mitral valve to the anterior valve dividing the mitral valve into two orifices. DOMV is classified into three types: complete bridge, incomplete bridge, and hole types, with complete bridges accounting for 15% of DOMV and the remaining two accounting for 85% (10, 11). This case was diagnosed as an incomplete bridge type. Moreover, DOMV rarely occurs as an isolated anomaly but is commonly combined with other malformations including atrioventricular septal defect, aortic stenosis, patent arterial duct, and tetralogy of Fallot (12, 13). Therefore, DOMV is usually detected in early childhood. Nevertheless, its prevalence and prognostic relevance in adulthood are unknown. Approximately half of DOMV patients are functionally normal and usually have no obvious signs and symptoms, thus predisposing them to underdiagnosis. In this study, DOMV was also found incidentally.

The ultrasound presentation of DOMV in adults is characteristic. Specifically, two left-right aligned orifices are visible at the level of the short axis of the parasternal mitral valve, showing the typical “spectacle sign.” In the left apical two-chamber view, two orifices exist in the mitral valve, exhibiting the typical “seagull sign” (14). Doppler examination reveals that each valve orifice obtains a corresponding diastolic flow. The present case demonstrates the superiority and importance of echocardiography for the accurate diagnosis of DOMV. In addition, echocardiography can also detect other combined malformations or secondary changes, which can provide a basis for clinical decision-making.

Treatment and prognosis of DOMV depend on the type and severity of mitral valve insufficiency. When it is an isolated DOMV, no management is required with a favorable prognosis (15). Surgical valve repair or replacement (16) should be performed when significant stenosis or insufficiency is present, according to the cause of the lesion and intraoperative exploration findings (17). If associated with other intracardiac or extracardiac malformations, the corresponding lesion should be treated and the DOMV should be managed appropriately. In our case, no significant changes in the structure and function of the heart were observed during the 2-year follow-up, making it unnecessary for this patient to receive treatment. Additionally, we do not intend to take a longer follow-up.

## Conclusions

Double orifice mitral valve does not necessarily have clinical symptoms and signs to draw clinical attention. Therefore, this case report aims to raise awareness of this rare entity and provide all patients with the opportunity for correct and timely diagnosis and appropriate management.

## Data availability statement

The original contributions presented in the study are included in the article/Supplementary material, further inquiries can be directed to the corresponding author.

## Ethics statement

Written informed consent was obtained from the patient for publication of this case report and any accompanying images.

## Author contributions

ZD and XW collected all the clinical data. QL and JL independently reviewed the data. ZD prepared the manuscript. JL and HL proposed the idea for this work. All authors contributed to the article and approved the submitted version.

## References

[1] Baño-Rodrigo A Van Praagh S Trowitzsch E Van Praagh R. Double-orifice mitral valve: a study of 27 postmortem cases with developmental, diagnostic and surgical considerations. Am J Cardiol. (1988) 61:152–60. 10.1016/0002-9149(88)91322-7 3276118

[2] Graham FJ Jenkins SMM. Isolated double-orifice mitral valve in octogenarian. Eur Heart J Cardiovasc Imaging. (2018) 19:957. 10.1093/ehjci/jey066 29688294

[3] Trowitzsch E Bano-Rodrigo A Burger BM Colan SD Sanders SP. Two-dimensional echocardiographic findings in double orifice mitral valve. J Am Coll Cardiol. (1985) 6:383–7. 10.1016/S0735-1097(85)80176-5 4019924

[4] Krisai P Wein B Kaufmann BA. Isolated double-orifice mitral valve: a case report. BMC Cardiovasc Disord. (2015) 15:172. 10.1186/s12872-015-0168-0 26681334 PMC4683737

[5] Liu S Ren W Ma C Yang J. Congenital double-orifice mitral valve in asymptomatic patients. Int Heart J. (2018) 59:213–5. 10.1536/ihj.17-033 29269712

[6] Lange M Bültel H Wichter T. Calcified mitral stenosis imitates a MitraClip(^®^) and forms a double orifice. Eur Heart J. (2018) 2:yty084. 10.1093/ehjcr/yty084 31020161 PMC6177079

[7] Bhamra-Ariza P Muller DW. The MitraClip experience and future percutaneous mitral valve therapies. Heart Lung Circ. (2014) 23:1009–19. 10.1016/j.hlc.2014.05.021 25035158

[8] Feldman T Kar S Rinaldi M Fail P Hermiller J Smalling R. Percutaneous mitral repair with the MitraClip system: safety and midterm durability in the initial EVEREST (Endovascular Valve Edge-to-Edge REpair Study) cohort. J Am Coll Cardiol. (2009) 54:686–94. 10.1016/j.jacc.2009.03.077 19679246

[9] Romano MMD Menardi AC Almeida-Filho OC Vicente WVA Evora PRB. Double-orifice mitral valve: an educational presentation. Braz J Cardiovasc Surg. (2019) 34:377–9. 10.21470/1678-9741-2018-0615 31310480 PMC6629215

[10] Wójcik A Klisiewicz A Szymański P Rózański J Hoffman P. Double-orifice mitral valve—echocardiographic findings. Kardiol Pol. (2011) 69:139–43. 21332053

[11] Pillai VV Karunakaran J. Repair of double orifice left AV valve (DOLAVV) with endocardial cushion defect in adult. Braz J Cardiovasc Surg. (2017) 32:338–40. 10.21470/1678-9741-2016-0034 28977206 PMC5613729

[12] Kowalik E Klisiewicz A Skrzypczyńska-Banasik U Hoffman P. Always look at both sides of the heart: a double-orifice mitral valve discovered in a young adult with repaired tetralogy of Fallot. Cardiol J. (2019) 26:204–5. 10.5603/CJ.2019.0045 31032874 PMC8086644

[13] Mouine N Amri R Cherti M. Unusual findings in secondary hypertension: double orifice mitral associated to aortic coarctation, bicuspid aortic valve, and ventricular septal defect. Int Arch Med. (2014) 7:14. 10.1186/1755-7682-7-14 24693935 PMC3976167

[14] Ciampani N Vecchiola D Silenzi C Costantini C Mazzanti M Iacobone G. The tensor apparatus in double-orifice mitral valve: interpretation of echocardiographic findings. J Am Soc Echocardiogr. (1997) 10:869–73. 10.1016/S0894-7317(97)70048-8 9356953

[15] Zalzstein E Hamilton R Zucker N Levitas A Gross GJ. Presentation, natural history, and outcome in children and adolescents with double orifice mitral valve. Am J Cardiol. (2004) 93:1067–9. 10.1016/j.amjcard.2004.01.015 15081462

[16] Zhu D Chen A Zhao Q. Surgical repair for isolated congenital double-orifice mitral valve. Eur J Cardiothorac Surg. (2011) 39:268–70. 10.1016/j.ejcts.2010.05.030 20619669

[17] Tani T Kim K Fujii Y Komori S Okada Y Kita T. Mitral valve repair for double-orifice mitral valve with flail leaflet: the usefulness of real-time three-dimensional transesophageal echocardiography. Ann Thorac Surg. (2012) 93:e97–8. 10.1016/j.athoracsur.2011.11.047 22450113

